# Conformational Dynamics of Response Regulator RegX3 from *Mycobacterium tuberculosis*

**DOI:** 10.1371/journal.pone.0133389

**Published:** 2015-07-22

**Authors:** Ashfaq Ahmad, Yongfei Cai, Xingqiang Chen, Jianwei Shuai, Aidong Han

**Affiliations:** 1 State Key Laboratory for Cellular Stress Biology, School of Life Sciences, Xiamen University, Xiangan, Xiamen, China; 2 Department of Physics, Xiamen University, Siming, Xiamen, China; Instituto de Tecnologica Química e Biológica, UNL, PORTUGAL

## Abstract

Two-component signal transduction systems (TCS) are vital for adaptive responses to various environmental stresses in bacteria, fungi and even plants. A TCS typically comprises of a sensor histidine kinase (SK) with its cognate response regulator (RR), which often has two domains—N terminal receiver domain (RD) and C terminal effector domain (ED). The histidine kinase phosphorylates the RD to activate the ED by promoting dimerization. However, despite significant progress on structural studies, how RR transmits activation signal from RD to ED remains elusive. Here we analyzed active to inactive transition process of OmpR/PhoB family using an active conformation of RegX3 from *Mycobacterium tuberculosis* as a model system by computational approaches. An inactive state of RegX3 generated from 150 ns molecular dynamic simulation has rotameric conformations of Thr^79^ and Tyr^98^ that are generally conserved in inactive RRs. Arg^81^ in loop β4α4 acts synergistically with loop β1α1 to change its interaction partners during active to inactive transition, potentially leading to the N-terminal movement of RegX3 helix α1. Global conformational dynamics of RegX3 is mainly dependent on α4β5 region, in particular seven ‘hot-spot’ residues (Tyr^98^ to Ser^104^), adjacent to which several coevolved residues at dimeric interface, including Ile^76^-Asp^96^, Asp^97^-Arg^111^ and Glu^24^-Arg^113^ pairs, are critical for signal transduction. Taken together, our computational analyses suggest a molecular linkage between Asp phosphorylation, proximal loops and α4β5α5 dimeric interface during RR active to inactive state transition, which is not often evidently defined from static crystal structures.

## Introduction

Microorganisms have been evolved with many sophisticated signal transduction systems to rapidly respond to various kinds of external and/or internal stimuli. Two-component systems (TCSs) have emerged as a major signal transduction pathway in microorganisms [1]. A typical TCS contains a membrane-integrated sensor histidine kinase (SK) and a response regulator (RR). A SK, which senses and interprets stimuli to activate its cognate cytoplasmic RR through phosphorylation, usually performs autokinase, phosphatase and phosphotransferase activities. RR mostly outputs as a transcription factor to alter expression level of specific set of genes [2, 3]. The phosphorylation is absolutely specific and conserved on a histidine residue in SK and an aspartic acid residue in RR, which is believed to initiate favorable conformational changes to promote RR dimer formation and stabilization [4, 5].

SenX3-RegX3 is an essential TCS for the survival and progressive infection of mycobacteria [6]. This TCS induces transcription of *phoA* and *pstS* genes in phosphate limiting environment [7] and also controls expression of several critical metabolic enzymes Ald, CydB and GltA1 in aerobic condition [8]. RegX3, which belongs to OmpR/PhoB family, comprises of two domains, N-terminal receiver domain (RD) and C-terminal effector domain (ED). The ED is a DNA-binding with a characteristic structure of winged helix-turn-helix (wHTH) and involved in transcriptional regulation upon phosphorylation of Asp^52^ in its RD [3, 9].

Structures of seven full-length OmpR/PhoB family members, MtrA, PrrA, DrrB, DrrD, PhoP, BaeR and RegX3 have been solved by X-ray crystallography [10–16]. All the structures, but not RegX3, are present in inactive state and therefore RegX3 becomes only a structure captured in active state, which is stabilized by five lanthanum ions used in crystallization condition. The La^3+^ ion that stabilizes the active site of RegX3 coordinates with Asp^9^ (β1α1 loop), Asp^52^, Met^54^ (β3α3 loop) and Glu^84^ (α4) where Asp^9^ and Asp^52^ prevent the steric clash. Moreover the position of La^3+^ ion almost coincides with Mg^2+^ in PhoB (PDB ID: 1ZES) [13, 17]. In CheY RR, trivalent cations are shown to bind tightly than divalent cations and suggested the importance of high positive charge that could further neutralize the repulsive effect of the carboxylate amino acids in the active site [18]. The active RegX3 has a unique domain-swapped dimer interface of α4β5α5 face in OmpR/PhoB family [17, 19]. The two signature switch residues Thr^79^ and Tyr^98^ form a so-called Y-T coupling or *gauche+* conformation, where Thr^79^ OH group points to the conserved Asp^52^ [3, 13].

Macromolecular structures determined by X-ray crystallography can be possibly restricted or even changed in crystallization conditions [20]. Neither cryo-EM nor NMR is sufficient to provide a complete and dynamic picture of molecules. Molecular Dynamics (MD) is able to simulate a macromolecular structure in a relaxed and physiological condition, which provides significant insights on protein functions at molecular level [21, 22]. The diverse structures of the full-length RRs indicate that a RR is capable to reorient both the RD and ED in response to phosphorylation-induced activation [23]; however, a comprehensive analysis of critical residues has not been reported. Here we took co-evolution analysis for OmpR/PhoB family, which was further supported by MD and normal mode analysis (NMA) using RegX3 as a model. We observed several key conformational changes in the local flexible regions of RegX3 phosphorylation site in addition to critical proximal residues, revealing a plausible molecular mechanism that transmits global transition signals between RD and ED upon phosphorylation.

## Results

### Inactive state of RegX3

The full-length RegX3 structure was solved in active conformation stabilized by domain swapping [13]. To obtain an inactive conformation of RegX3s, we used MD simulation for 150 nanoseconds (ns) in explicit solvent condition and analyzed the convergence of conformational changes of RegX3 among MD trajectories with Principal Component Analysis (PCA), which analyzes total variance or mean-square displacement of atomic positional fluctuations in eight dimensional components (PCs). More than 60% of the variance was captured by the first two principal components (PCs), representing five major clusters of relatively stable structures (S1 Fig).

**Fig 1:**
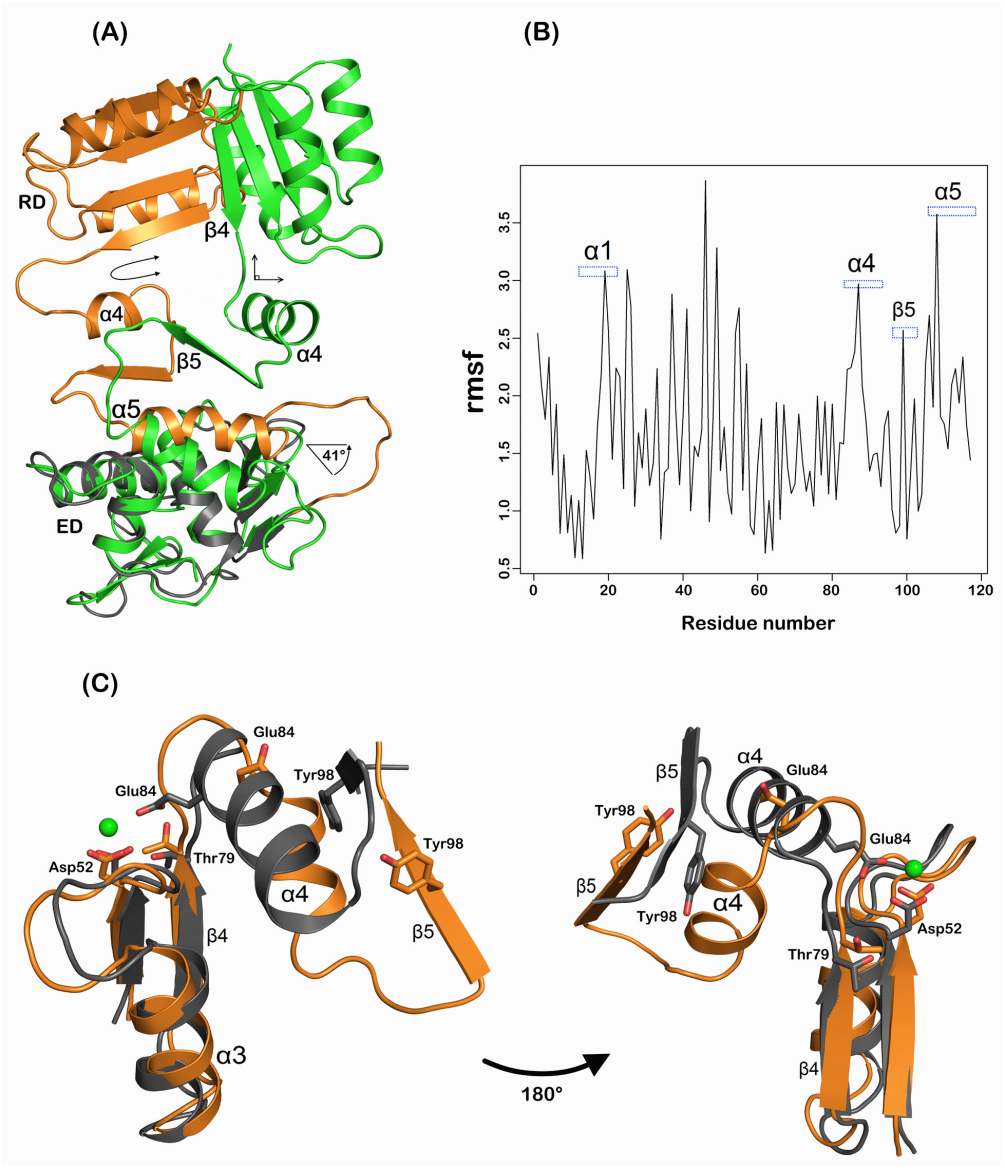
Conformational switch of RegX3 active Pocket in MD simulation. (A) Structural alignment of simulated RegX3s (colored in orange) and original active RegX3 (colored in green). The alignment was based on core folding of ED. α4β5α5 face elements are labeled. Rotation degree of α5 is indicated with arrow in the lower half of the structure. Perpendicular and parallel switch for β4α4 are marked in the upper half of the structure. (B) High mobility of RegX3 residues based root-mean-square fluctuation (rmsf). (C) Active pocket stabilized by key residues together with a lanthanum ion in the active conformation of RegX3 in comparison with simulated RegX3s. The alignment and color scheme are the same as panel A. Coupled residues Thr^79^ and Tyr^98^ together with other important residues around active site are labeled with sticks. The green sphere represents Lanthanum ion.

The simulated RegX3s adopts a reasonably compact conformation, which is clearly different from its original active state (rmsd of 12.5 Å for all Cα atoms) (Fig 1A and S2 Fig). Globally, the ED swings 174° toward the RD with α4 rotation of 51.6°, resulting in a compact conformation resembling MtrA and PrrA, when compared with the active RegX3 (S2A Fig). Partial helical unwinding and rotation of α4 helix change its original perpendicular positioning to a position parallel to the RD of RegX3s as in PhoB, TorR, MtrA and YycF. The hydrophobic patches Val^88, 89^ and Leu^93^ are protected by β4 and β5. In addition, the helical unwinding of α4 leads two amino acids extension of loop β4α4. Meanwhile, α5 rotates 41° in the same direction as α4, completing the α4β5α5 face in an almost parallel conformation to both domains (Fig 1A). During the simulation, α4β5α5 and the N-terminal region of α1 appear to have significant conformational changes (Fig 1B and S3 Fig). In contrast, the ED keeps a rigid conformation as active RegX3 during simulation (rmsd of 1.2 Å for 96 Cα atoms) (S2C and S2D Fig). The core α/β fold of the RD is even more rigid (rmsd of 0.76 Å for 74 Cα atoms when aligned to active RegX3), which is also well conserved in inactive DrrD (PDB ID, 1KGS), BaeR (PDB ID, 4B09), MtrA (PDB ID, 2GWR) and PrrA (PDB ID, 1YS6) (rmsd of 0.9–1.5 Å).

**Fig 2:**
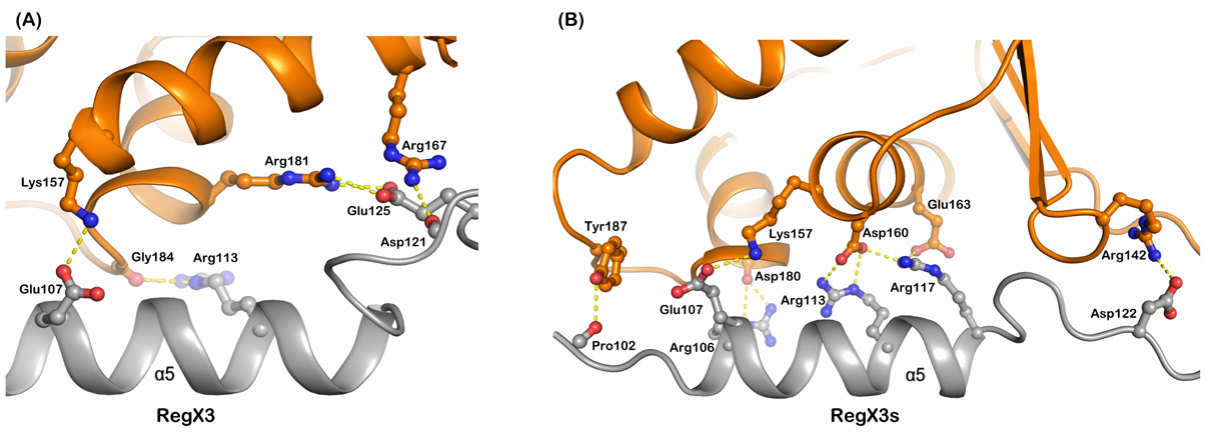
Interdomain interface between receiver and effector domains. Interdomain interactions between RD and ED domains of original active state (A) and simulated inactive state RegX3 (B) are shown in cartoon. The top half of each panel is from the ED in orange; the lower half is α5 and its adjacent loop in grey from the RD. Sticks represent all interaction residues with hydrogen bonds in yellow dotted lines.

Compact conformation of RegX3s is appropriate with tighter interdomain interactions than the active RegX3, which are mediated mainly by α5 as well as loop β5α5 of 11 residues in RD with α7 and loop α7α8 (transactivation loop) in ED (Fig 2). Asp^122^ interacts with Arg^142^ in RegX3s and Asp^121^ with Arg^167^ in RegX3. Glu^107^ keeps the same interaction with Lys^157^ in both states. Arg^113^ interacts with Asp^160^ in RegX3s but with Gly^184^ in RegX3. The interdomain interface of inactive RegX3s is 868 Å^2^, comparable with other inactive RRs, for examples, MtrA (738 Å^2^), PrrA (820 Å^2^), DrrB (751 Å^2^) and DrrD (245 Å^2^).

More importantly, the signature residue Tyr^98^ of the α4β5α5 face adopts an outward conformation as in inactive states of other RRs, while it flips without any rotameric changes to its coupling residue Thr^79^ in original active RegX3. The side chain of the second signature switch residue Thr^79^ adopts a new conformation that faces against the active pocket (Fig 1C). Together, we concluded that RegX3s has adopted a putative inactive conformation of RegX3 generated from MD simulation.

### Conformational dynamics of loops

Several loops surrounding the active site were proposed to be vital for RR phosphorylation and global conformational changes [21]. In order to examine their dynamics in RegX3, the trajectories for β1α1, β3α3 and β4α4 loops from both active and inactive states were assembled and analyzed in LigPlot^+^ and *Bio3d*.

**Fig 3:**
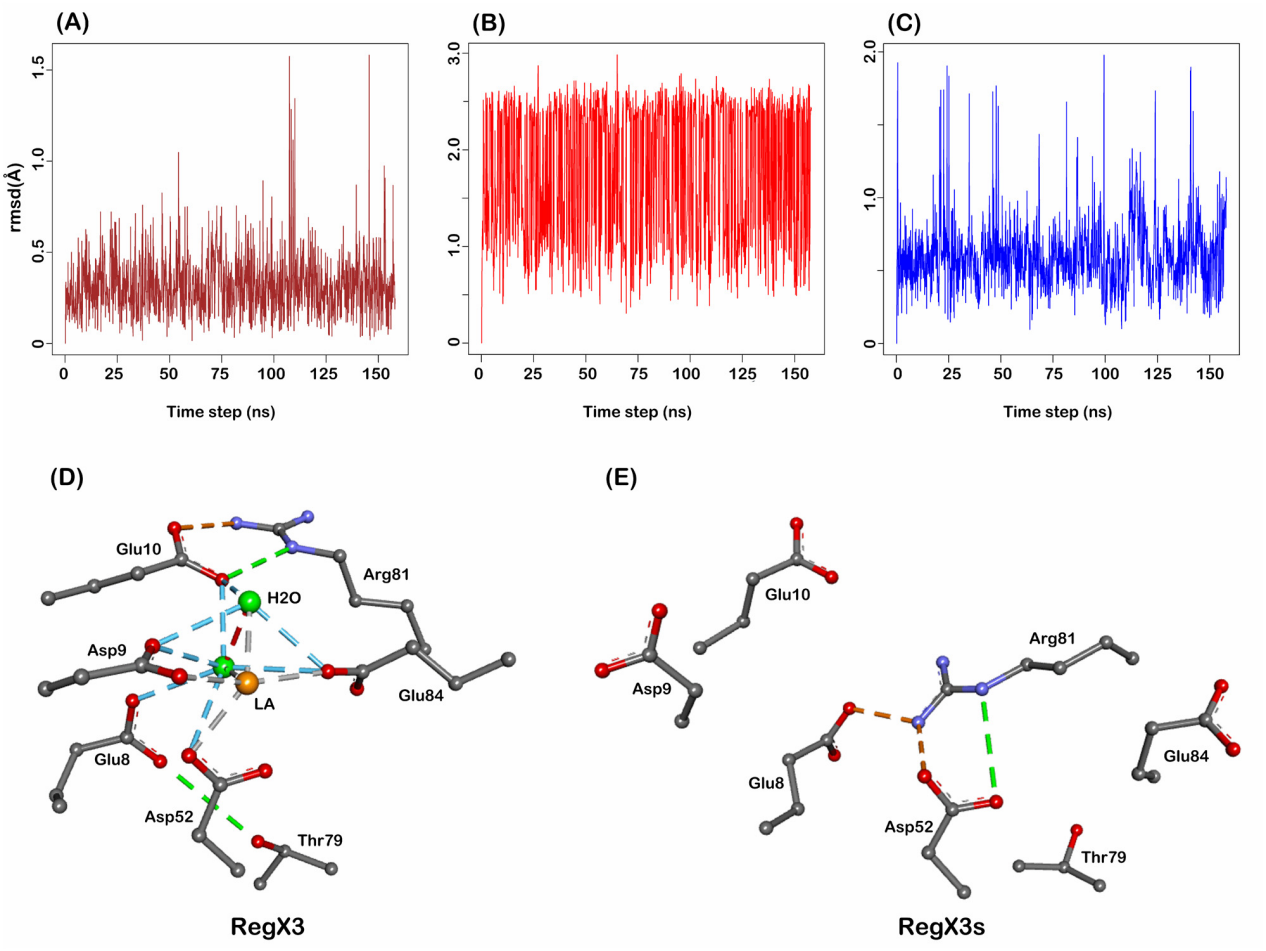
Dynamics of β1α1, β3α3 and β4α4 loops. (A-C) rmsd deviation plots of β1α1, β3α3 and β4α4 loops of RegX3 during 150 ns simulation. X-axis is time step and Y-axis indicates rmsd deviations. Interaction networks of these loops are explicated in active state (D) and inactive state (E). Residues are shown in ball and sticks. Brown and green spheres indicate La^3+^ ion and water molecule, respectively. All interactions are highlighted in dashed lines.

Loops β1α1 (amino acids 8–10) and β4α4 (amino acids 79–82) stabilize the active RegX3 conformation through interactions with trivalent ions [13]. Loop β1α1, composed of two glutamic acids and one aspartic acid, exhibits the least conformational fluctuation (rmsd of 0.5 Å for 3 Cα atoms) during active to inactive transition (Fig 3A). In contrast, loop β4α4, as a connection between α4β5α5 face and the RD core region, shows a slightly larger conformational change (rmsd 0.8 Å for 4 Cα atoms) (Fig 3C). Both loops form an interaction network with La^3+^ ion and water through Arg^81^, Glu^84^, Glu^10^ and Asp^9^ together with Asp^52^ in active RegX3 (Fig 3D). However, significant conformational shift of loop β4α4 in inactive RegX3s renders Arg^81^ to make two hydrogen bonds directly with Asp^52^ and projects Asp^9^ and Glu^10^ out of the phosphorylation site (Fig 3E). During transition the observed dynamics of these loops are in good agreement with previously published data (20, 36).

Taken together, these data from the MD simulation suggest that the loop β4α4 extended from rotation and partial helical unwinding of α4 causes Arg^81^ to establish a completely different interaction network in inactive RegX3s. It is interesting to note that loop β3α3 (amino acids 53–59) exhibits the most conformational fluctuations (rmsd of 2.0 Å for 7 Cα atoms) among these three loops during the active-inactive transition (Fig 3B), but it does not appear to change the interaction network in phosphorylation site (Data not shown).

### Conformation transition network

**Fig 4:**
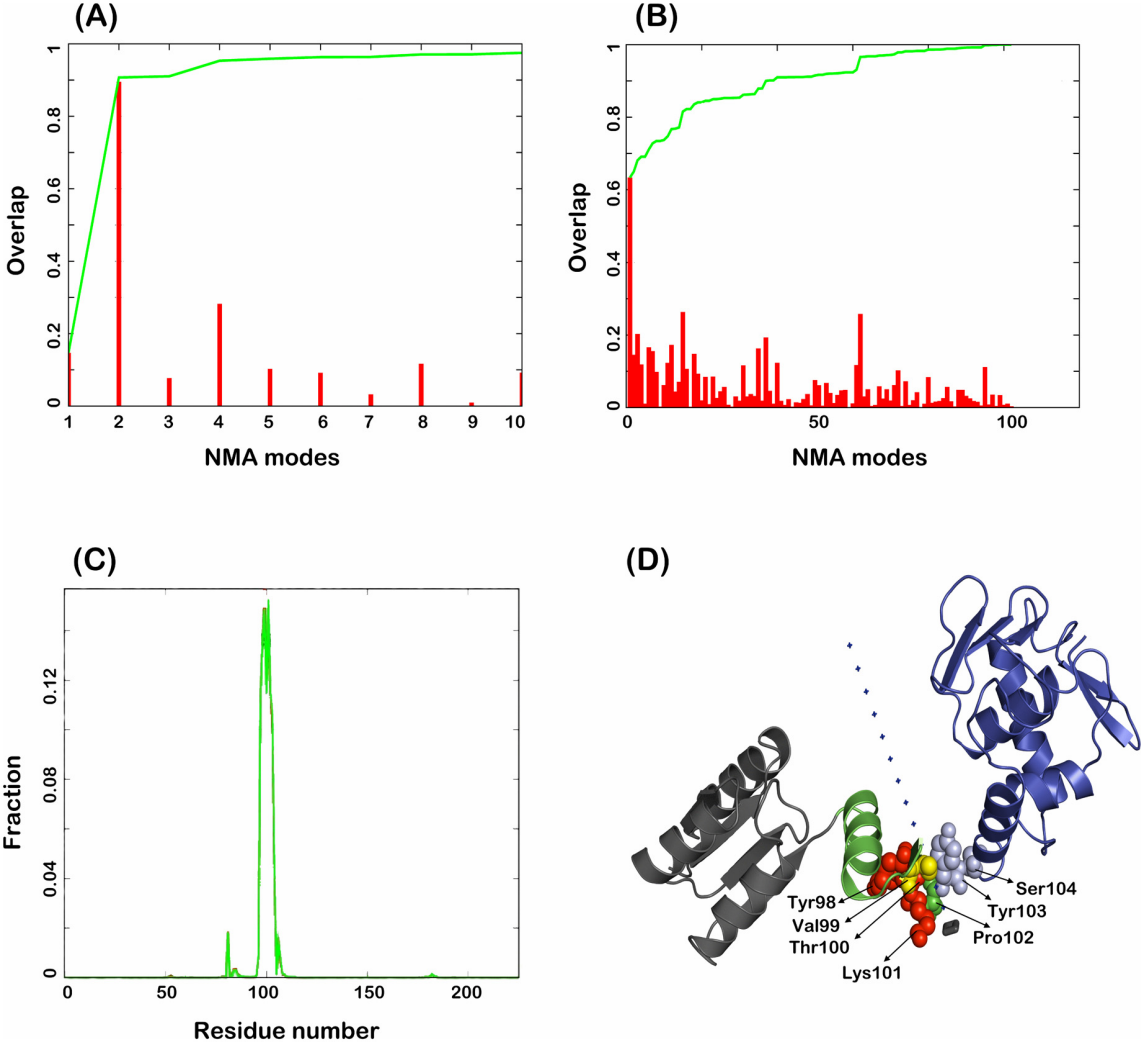
Normal model analysis for conformational dependent regions of RegX3. (A) Overlap score of all non-zero low frequency modes between active and inactive state structures. (B) Overlap score of non-zero ENM modes based on the contribution of pocket residues of α4β5α5 face to the overall changes of full-length RegX3. (C) Contribution fraction (Y-axis) of residues to low frequency NMA modes in pocket residue analysis. (D) Key structural region with the nature of individual residues contributes to conformational changes during active to inactive transition of RegX3. ED is colored in grey and RD in blue. Helix α4 is colored in green and hot spot residues are in sphere models. The direction of an arrow represents the hinge motion of blue (ED) towards black (RD).

Low frequency normal mode analysis (NMA) of elastic network model (ENM) is a preferable approach to capture long-range conformational change, which is otherwise very expensive for MD [24–26]. The low frequency NMA modes depict the functional relevance. The estimated collective parameters of motions were calculated for the active to inactive transition of RegX3. The collective overlap score for the first 10 modes was 0.95, which was dominated by mode 2 (the core of 0.84), indicating that the transition of RegX3 structures is functionally relevant (Fig 4A).

The NMA non-zero mode 2 revealed a closure rotation of RD towards ED using a hinge region α4β5, suggesting that α4β5 effectively controls the fluctuation of RegX3. To investigate dependency of the total overlap on α4β5α5 face during the active to inactive transition, we performed NMA again using α4β5α5 face as a pocket region. The initial modes were dominant and scored approximately 0.64, suggesting dependency of the α4β5α5 face in RegX3 conformational transitions (Fig 4B). Consistently, the coherent nature of the pocket residue analysis further indicated an importance of the α4β5 region but not α5 in RegX3 function (Fig 4C). It is interesting that this analysis also showed a relatively small signal for Asp^82^. To further refine α4β5 as a critical region in transition, we performed a hot-spot analysis. Residues Tyr^98^ to Ser^104^ in RegX3 exhibited high σω value in the overall elastic distortion, and thus considered as the “hot-spot” residues (Fig 4D).

These hot-spot residues may likely be important to modulate global dynamics between active and inactive states of RRs in long-range conformational communications. Tyr^98^ has been shown to play a key role in allosteric transition of OmpR/PhoB family whereas Lys^101^ interacts with the side chain of Thr^79^ only in active RR [3, 13]. Taken together, our NMA analysis indicates that residues Tyr^98^ to Ser^104^ of α4β5 region are the most important in RegX3 conformational switch.

### Coevolution analysis

**Fig 5:**
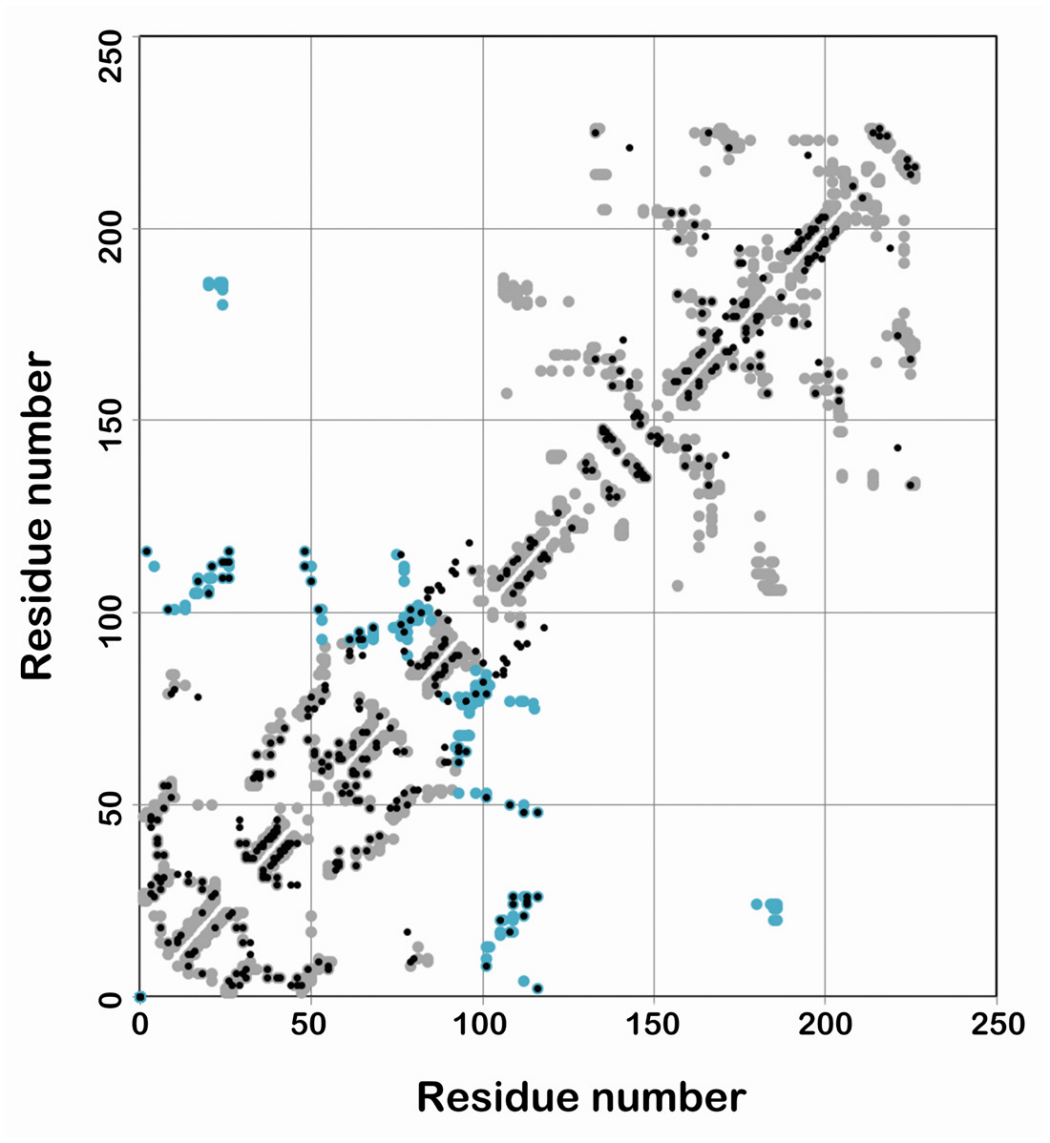
Coevolution analysis of RegX3. Contact map of coevolved residues in OmpR/PhoB family. Axes are residue numbers. Grey spots represent contacts within the same monomer, while black spots are predicted inter-monomeric contacts. The blue spots highlight those coevolved pairs in dimeric interface listed in Table 1.

We next performed a coevolution analysis to search for critical residues connecting the dimeric interface and global conformational changes. We collected 29,283 members of OmpR/PhoB family and analyzed them using a program GREMLIN, which constructs a global statistical model, assigns probability to each residue and calculates all evolving positions (Fig 5). The top 23 pairs of coevolved residues involved in dimeric interface were selected within the cutoff distance of 5 Å (Table 1).

**Table 1:** Coevolved residues of dimer interface of OmpR/PhoB family calculated by GREMLIN: Listed are the interacting residues with their respective numberings and topology. Italicized pairs indicate coevolved inter-monomeric contacts of RegX3 but intra-monomeric contacts in other OmpR/PhoB family members. Bold representation highlights those dimeric interactions conserved in all OmpR/PhoB family members. Contact pair with asterisk symbol satisfies all available active RR structures in this family but not RegX3.

Observed_score	Partner 1	Partner 2	Topology
2.026	*Leu20*	*Ala105*	*α1- α5*
2.162	Lys87*	Glu107*	α4-α5
1.73	*Ile48*	*Leu112*	*β3-α5*
1.715	*Leu21*	*Leu112*	*α1- α5*
**1.526**	**Asp97**	**Arg111**	**β5-α5**
1.441	*Arg68*	*Asp96*	*α3- β5*
1.354	*Glu24*	*Ile109*	*α1- α5*
1.315	*Thr61*	*Leu93*	*α3- α4*
1.206	*Phe26*	*Arg113*	*L2-α5*
1.155	*Ile48*	*Leu116*	*β3-α5*
0.983	*Leu17*	*Leu108*	*α1-α5*
0.955	*Phe26*	*Leu109*	*L2-α5*
**0.896**	**Glu24**	**Arg113**	**α1- α5**
**0.869**	**Ile76**	**Asp96**	**β4- β5**
0.866	*Asp82*	*Thr100*	*L7- β5*
0.834	*Lys65*	*Leu93*	*α3- α4*
0.831	*Glu8*	*Lys101*	*L1- β5*
0.826	*Thr2*	*Leu116*	*β1-α5*
0.801	*Leu50*	*Leu108*	*β3-α5*
0.79	*Thr79*	*Lys101*	*β4- β5*
0.777	*Cys64*	*Ala95*	*α3- β5*
0.754	*Asp52*	*Lys101*	*β3- β5*
0.722	*Phe26*	*Leu116*	*L2- α5*

**Fig 6:**
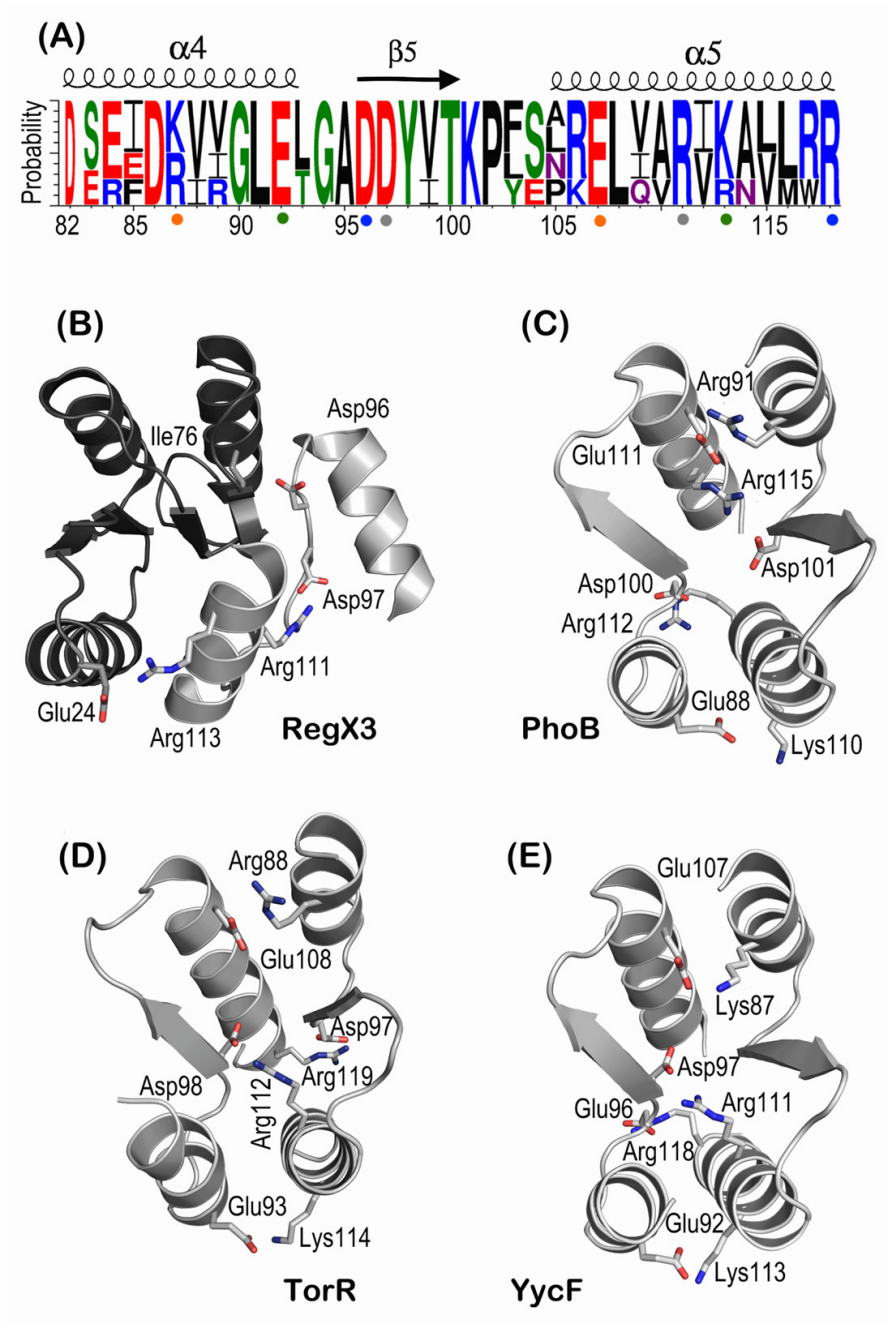
Coevolved residues in dimeric interface of OmpR/PhoB family. (A) Sequence conservation of the α4β5α5 dimeric interface of RegX3, PhoB, TorR and YycF in OmpR/PhoB family. Residues are numbered according to RegX3 and colored based on chemistry: acidic in red, basic in blue, hydrophobic in black, polar in green and neutral in purple. Coevolved pairs are under-dotted with the same colors. (B-E) Active state structures of RegX3 (2OQR), TorR (1ZGZ), PhoB (1ZES) and YycF (1NXP) in OmpR/PhoB family with coevolved residues in dimeric interface highlighted in sticks. It is noteworthy that RegX3 forms a complete dimeric structure with swapped domains that are colored in light and dark greys.

We then compared these coevolved residues in RegX3, PhoB (1ZES), TorR (1ZGZ) and YycF (1NXP) in OmpR/PhoB family. Residues of 19 pairs were involved only in stabilization of RegX3 dimer and not of PhoB, TorR and YycF, which were therefore excluded from our analysis (Table 1, italicized). The remaining four pairs are conserved in dimer interfaces of active PhoB, TorR and YycF (Fig 6). Lys^87^-Glu^107^ pair does not contribute to RegX3 dimerization although it surely is one of key residues in PhoB, TorR and YycF. In contrast, Asp^97^-Arg^111^ pair is an important player in RegX3 since it stabilized conformation of β5 and α5 by a salt bridge [14]. Therefore, Glu^24^-Arg^113^, Asp^97^-Arg^111^ and Ile^76^-Asp^96^ were critical residues to connect the dimeric interface with global conformational changes during active to inactive transition of RegX3.

### *In silico* mutagenesis

To further study these coevolved residues on RegX3 conformational changes, we subjected the active RegX3 with a series of alanine mutations for residues Glu^24^, Ile^76^, Asp^96^, Asp^97^, Arg^111^ and Arg^113^ to 5 ns MD simulation. The original active RegX3 was also set up to generate RegX3-5ns as a control. Trajectories from these MD simulations were assembled by Nosé-Hoover constant pressure and temperature (NPT) system, and analyzed by PCA for variances of the first 2 PCs, leading to estimation of mutational effects for these single or double mutations (S4 Fig). RegX3-5ns maintains the same trajectory of α4 and α5, but β5 and α5 parallel shift about 8 Å (S5A Fig). In contrast, mutant mRegX3(96) shows 31° rotation and 13 Å parallel shift of β5 while mRegX3(76,96) has nearly 120° rotation of β5 and α5 in addition to 46° rotation of α4 (S5B and S5C Fig). All secondary structures of α4β5α5 have as large as 30–75° rotations in mRegX3(97), mRegX3(111) and mRegX3(97,111), which are not observed in RegX3-5ns (S5D, S5E and S5F Fig). More dramatically, α4β5α5 all switch into perpendicular positions in mRegX3(113) and shift 68° in addition to a large rotation in mRegX3(24,113) (S5G and S5H Fig). It is noteworthy that Ile^76^ is important to mediate domain-swapped dimer only in RegX3 but not for other OmpR/PhoB family [13]. Together, all these mutations seem to release constrains on conformation of α4β5α5 region compared with wild-type RegX3 in our MD experiments, suggesting that these key residues identified from the coevolution analysis are indeed important for RegX3 structural dynamics.

**Fig 7:**
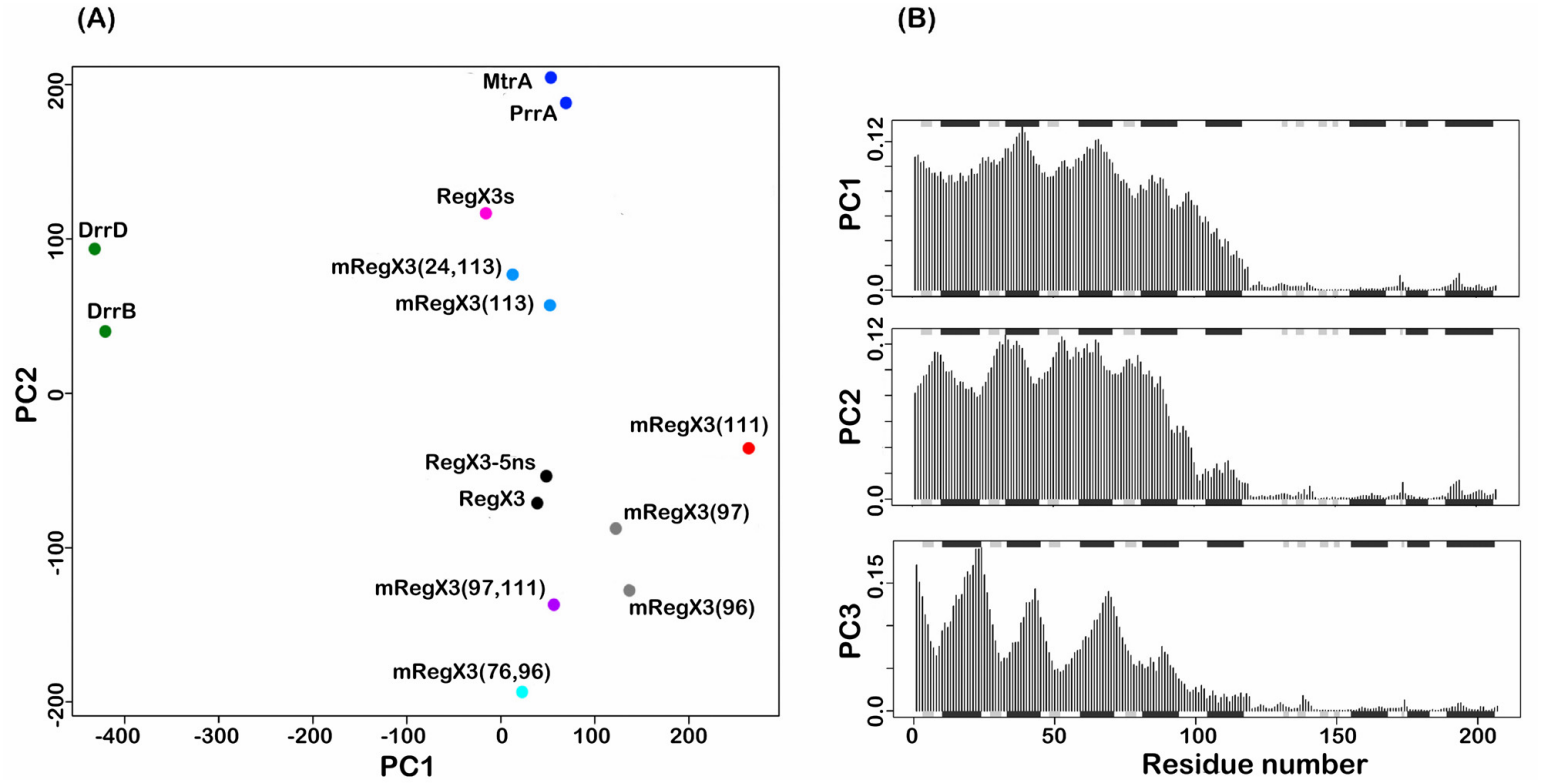
In silico mutagenesis analysis of RegX3. (A) Simulated structures of active RegX3 with introduced mutations are analyzed by PCA. RegX3s and RegX3-5ns used as a control are those simulated structures after 150 ns and 5 ns, respectively. mRegX3 indicates RegX3 mutant simulated for 5 ns with numbers in parenthesis for alanine mutation of specific sites. DrrB and DrrD are representative inactive and open RR structures with exposed DNA recognition helices. MtrA and PrrA are representative inactive and compact structures with buried DNA recognition helices. Structures are grouped by color dots. (B) The frequency distribution of individual residues contributing to all RegX3 structures on PC spaces. Y-axis indicates the observed frequency and X-axis represents the residue numbers of RegX3. Grey and black color regions on the X-axis indicate β sheets and α helices whereas the white spaces in between indicate loop regions.

We then analyzed these structures of RegX3 mutants for global conformational changes with OmpR/PhoB family members using *Bio3d* [27] and differences in these structures were presented in PCA planes (Fig 7A). DrrB and DrrD form one inactive group with an extended conformation. The simulated RegX3s is clustered next to PrrA and MtrA as another inactive group with a compact conformation, whereas RegX3 clusters with RegX3-5ns, retaining an active group. The mutants mRegX3(96), mRegX3(97), mRegX3(111), mRegX3(97,111) and mRegX3(76,96) are clustered near the active group. Interestingly, mRegX3(113) and mRegX3(24,113) appear to attain a transitional conformation between the active and inactive groups.

Together, our *in silico* mutagenesis experiments indicates different effects of these coevolved residues on conformational changes of RegX3. The conservation of these coevolved residues depicts their importance for OmpR/PhoB family. Interestingly, the residues responsible for these structural distributions are in N-terminal RD rather than C-terminal ED (Fig 7B), further signifying the dominant role of RD in regulating the global conformation of a RR.

## Discussions

In OmpR/PhoB family, phosphorylation actuates dimerization through two possible pathways: rearrangement of RD and ED domains that overcomes dimerization inhibition and subtle conformation changes of some critical residues at the dimer interface [28, 29]. However, connections between these two pathways in conformational dynamics of a RR have not been thoroughly investigated. In this study we generated an inactive state of RegX3, a RR in OmpR/PhoB family through 150 ns MD and compared it with its original active state by a variety of computational analyses. Further analysis by coevolution identified several critical interaction residues, which were fully supported by *in silico* mutagenesis. All these portray a coherent picture of molecular dynamics of OmpR/PhoB family during active to inactive transition.

### Conformational dynamics of RegX3

Inactive state of RegX3 generated from 150 ns MD has a compact global conformation and typical rotameric conformation of Tyr^98^ and Thr^79^ as other inactive RRs (Figs 1 and 2). A hinge motion of α4β5 region bears a sufficient flexibility for RegX3 to mediate the active to inactive transition process (Fig 4). Indeed, phosphorylation of NtrC, FixJ, DctD, Spo0F and PhoB has been shown to induce conformational changes mainly in loop β4α4 and helix α4 [30–34]. α4 is also structurally unstable in other RRs such as CheY, DrrD and BaeR [10, 11, 35]. In RegX3, helix α4 adopts a perpendicular position that is closer to DrrD and BaeR, however in inactive state it rotates 51.6° occupying a parallel position to the core RD as in PrrA and MtrA [12, 15]. The rotation of α4 is accompanied by β4α4 loop that subsequently exposes Thr^79^ and partially Tyr^98^ as in inactive DrrD (Fig 1C). Such inactive rotameric state of Tyr^98^ has been suggested as the third state for inactive RRs [17].

Loop β4α4 is shown with more dramatic conformational changes than loop β1α1 and β3α3 during active to inactive transition, which breaks down the interaction network built by Thr^79^, Glu^8^, Glu^10^, Asp^52^ and Glu^84^ together with Arg^81^. Consequently, the conserved Arg^81^ replaces its interaction partner with Glu^8^ and Asp^52^ to achieve the inactive state (Fig 3). On the other hand the re-positioning of Arg^81^, Asp^9^ and Glu^10^ in inactive state allows 5.7° rotation of N-terminal helix α1 (S3 Fig). A slow motion of helix α1 has been shown critical for interactions with KinA, Spo0B and RapB [36]. In addition, these loops stabilize the phosphorylatable Asp^52^ in active state of RegX3, which is important in dephosphorylation for specific recognition by sensor histidine kinases [37, 38]. The re-orientations of Thr^79^, Arg^81^ and Glu^84^ render α4 in a parallel position to the core RD, which is further supported by the 41° axial rotation of α5 that secures a compact conformation of RegX3s through new contacts of Arg^113^ and Asp^160^, Arg^106^ and Asp^180^ and Arg^117^ and Glu^163^ on inter-domain interface (Fig 2B). At sub-family level of RRs (OmpR/PhoB), Arg^113^ is an evolutionary conserved residue of dimeric interface (Fig 6A) and interacts with Glu^92^ in active state dimer.

The dynamic relevant hot-spots residues may probe specific responses to induce local changes that mediate signal transmission. A cluster of seven hot spots (Tyr^98^ to Lys^101^) with high δω values, containing C terminal half of β5 and its adjacent loop, are defined as most critical residues in active-inactive transition. Consistently, Tyr^98^ and Lys^101^ have been shown important to maintain a stable environment for phosphorylation site in OmpR/PhoB family [13, 39–41]. The α5 is less important but may possibly involve in interdomain interaction of RegX3 to form a compact conformation (Fig 2).

### Critical residues on dimer interface

Last decade has seen considerable effect to study residue-residue contacts by covariation from aligned sequences using direct-coupling information and inverse-covariance, e.g. Direct Coupling Analysis (DCA) and Protein Sparse Inverse COVariance (PSICOV) [42, 43]. GREMLIN program recently developed by Baker and his colleague obtains model parameters from conditional correlations with fewer sets of protein sequences using a frame work of pseudo-likelihood integrated with structural information [44]. The authenticity of GREMLIN was tested using a crystal structure of VicK [45] and its homologues (data not shown), which demonstrated its efficiency to capture co-evolutionary signals in both active and inactive states of sensor histidine kinases as DCA [42, 46].

The α4β5α5 region is important for transition from inactive to active state [47, 48]. The specificity of homo- and hetero-dimer interface in OmpR/PhoB has been demonstrated by Förster resonance energy transfer (FRET) analysis [49]. Our coevolution analysis suggested that the specificity of dimeric interface in OmpR/PhoB family is evolutionarily optimized (Fig 6). We identified 23 coevolved pairs involved in the dimer interface with top scores (Table 1). Three pairs of Glu^24^-Arg^113^, Asp^97^-Arg^111^ and Ile^76^-Asp^96^ were selected as critical residues for RegX3 after excluding all intra-monomeric contacts. Mutations of these residues altered the dynamics of global conformation of RegX3 (Fig 7 and S5 Fig). Consistently, Arg^113A/E^ mutation (Arg^111^ in RegX3) abolished the dimer formation of active state PhoP *in vivo*. Single mutation Arg^115D^ dramatically decreased PhoB DNA binding and transcriptional activities, which were partially rescued by compensatory mutation Asp^101R^ (Asp^96^ and Arg^111^ in RegX3) [50]. Altogether, this data further substantiate that α4β5α5 face is critical for RR conformational change and dimerization during active to inactive transition.

## Summary

RRs are sensitive to phosphorylation-induced conformational changes. Phosphorylated RRs are stabilized by divalent ions like Ca^2+^ or Mg^2+^, which utilize binding sites from β1α1 and β4α4 loops. In active state these proximal loops are in stretched conformation and face the C-terminus of β3. The absence of divalent and phosphate ions from the active pocket remodeled β1α1 loop from a stretched to a relaxed conformation resulting the outward orientation of Glu^10^ and Asp^9^ that is supported by Arg^81^ from β4α4 loop. Arg^81^ breaks its contacts with the loop β1α1 to establish a new interaction network with the phosphorylatable aspartate, which possibly promotes an outward movement of N-terminal helix α1. The hot-spot cluster of α4β5 is most critical in maintaining the active/inactive conformation of a RR. All in all, these studies suggest a general working model for OmpR/PhoB family in which the α4β5 region with several coevolved residues together with Arg^81^ are pivotal for global and local structural dynamics that govern the active to inactive conversion induced by RR phosphorylation state.

## Methods

### Coevolution analysis

A total of 29,283 sequences (The alignment file is available upon request) of all full length response regulators were gathered and an alignment was constructed using HHblits server [51]. The aligned sequences [X_1_, X_2_, X_3_,..,X_p_] with positions 1:p, were filtered by removing those sequences with >90% identity and more than 75% gaps. A program GREMLIN was used to extract all possible coevolution signals. The reduction of w_ij_ matrices to single values reflects the total strength of the coupling between positions i and j. We first computed s_ij_, their vector 2-norm (the square root of the averages of the squares of the individual matrix elements) and corrected differences in s_ij_ due to sequence variability using the row and column averages of these values:

$$S_{ij}^{corr}=S_{ij}-\frac{<S_{\text{kj}}{>}_{\text{k}}<S_{ik}{>}_{\text{k}}}{<S_{kl}{>}_{\text{kl}}}$$

The brackets represent averages taken over the indices outside the brackets in a manner similar to Average Product Correction (APC) [52]. The normalized coupling strength or “scaled score”, ncs_ij_, was computed by dividing the S_ij_^corr^ value by the total average of the top 3L/2 S_ij_^corr^ values (since there are roughly 3L/2 contacts for a protein of length L [44].

### Gremlin model construction from paired alignments

GREMLIN constructs a global statistical model, and assigns a probability to every amino-acid in an alignment:

$$\text{p}\left({\text{X}}_{1},{\text{X}}_{2}\ldots ,\;{\text{X}}_{\text{p}}\right)=\;\frac{1}{\text{z}}\text{exp}\left(\Sigma_{1}^{\text{p}}\left[{\text{v}}_{\text{i}}\left({\text{X}}_{\text{i}}\right)+\Sigma_{\text{j}=1}^{\text{p}}{\text{w}}_{\text{i},\text{j}}\left({\text{X}}_{\text{i}},{\text{X}}_{\text{j}}\right)\right]\right)$$

where *v*_*i*_ are vectors encoding position-specific amino acid propensities and w_ij_ are matrices encoding the coupling of amino acids between positions i and j. All these parameters are determined by maximizing the regularized pseudo-likelihood of the sequence alignment as described in [44, 53].

$$\text{v},\text{w}=\text{argmax}\Sigma_{1}^{\text{N}}\;\Sigma_{1}^{\text{p}}\text{logP}({\text{X}}_{\text{i}}|{\text{X}}_{1}\ldots {\text{X}}_{\text{i}-1}{\text{X}}_{\text{i}+1}\ldots {\text{X}}_{\text{p}}\text{)}+\text{R}\left(\text{v},\text{w}\right)$$

Where each summation is a conditional distribution capturing the probability of an amino acid at particular position in context of protein sequences. Regularization term R(v,w) is used to prevent over-fitting.

### Molecular dynamics

NAMD *2*.*10* (Beckman Institute, University of Illinois) was used to generate ensemble trajectories of RegX3 [54]. All individual MD simulations were performed in explicit solvent system with constant parameters. Protein structure files (psf) were generated using CHARMM36ff [55]. The whole system was neutralized by NaCl and solvated in an 8 Å cubic box. The default configuration settings were used with 1/Å^3^ PME grid density. Short-range, non-bonded interactions were calculated using a distance cut-off of 12 Å. The system was minimized for 1000 steps and simulated in the NPT ensemble (T = 310 K, P = 1 atm) with periodic boundary conditions having full particle-mesh Ewald electrostatics. Ligplot+ was used to analyze intra-protein contacts in active and inactive states of RegX3 [56]. Mutations were introduced into RegX3 structure using Modeller [57]. All structural illustrations were prepared in Pymol (DeLano Scientific LLC).

### Principal Component Analysis

To compare different MD conformation, we performed principal component analysis (PCA) using a package Bio3d [27]. PCA is a multivariate technique, which minimizes the maximal variance of the data to two or three dimensions through examining inter-conformer relationships, based on the covariance matrix, C, with two elements *i* and *j* originates from the Cartesian coordinates of the all superposed structures.

$$C_{ij}=\;\langle \left(X_{i}-\langle X_{i}\rangle \right)\left(X_{j}-\langle X_{j}\rangle \right)\rangle$$

Where X is the mass weighted Cartesian coordinate of an N-particle system and *C*_*ij*_ represents an average of all sampled structures. The C matrix can be diagnolized with orthonormal transformation matrix R:

$$R^{T}CR=diag\left(\lambda_{1},\lambda_{2},\ldots .\lambda_{3N}\right)$$

T is transpose of R and λ_1_ ≥ λ_2_ ≥, …. ≥ λ_3N_ indicates eigenvalue. To obtain the principal components q_i_(t), *i* = 1,….3N, the trajectories can be projected on the eigenvectors in the columns of R.

$$q=\;R^{T}\left(x\left(t\right)-<x>\right)$$

In the direction of principal mode, the eigenvalue λ_1_ is the mean square fluctuation. In PCA the first few PCs normally show global motions and contain the largest root mean square fluctuation.

### Elastic Network Model (ENM)

Elastic potentials of Cα coordinates of both active and inactive RegX3 as elastic bodies were constructed with a force constant of C indicating pair wise interaction of Cα atoms within *R*_c_ cut-off distance. The resultant potential energy of the elastic network is expressed as:

$$\text{E}\left({\text{x}}^{\to }-{\text{x}}_{{}^{\circ}}^{\to \;}\right)\;=\;\frac{1}{2}\underset{d_{ij}^{{}^{\circ}}<RC}{\sum }\text{C}{\left({\text{d}}_{ij}-{\text{d}}_{ij}^{{}^{\circ}}\right)}^{2}$$

Where x is a 3*N*-dimmentional vector representing Cartesian coordinates of RegX3 Cα atoms and x_o_ is a corresponding vector indicating Cα positions in RegX3 structure. d°_ij_ and d_ij_ represent the corresponding distance between two structures and two Cα atoms at positions i and j, respectively.

The above equation of potential energy calculation for elastic network is expanded to its second order by computing its Hessian matrix H, which best describes large amplitude motions in a protein for low frequency normal modes [58]:

$$E(\delta x^{\to })\approx \;\frac{1}{2}\delta x^{\to T}.\;H.\delta x^{\to }$$

MENM potential functions of RegX3 were calculated as Best et al [59]:

$$E(x^{\to })=-\beta^{-1}1n\left[e^{-\beta \left(E1\left(x^{\to }-x_{1}^{\to }\right)+\in 1\right)}+e^{-\beta \left(E2\left(x^{\to }-x_{2}^{\to }\right)+\in 2\right)}\right]$$

Where Ɛ1 and Ɛ2 indicate energy off-sets and *β* is *T*_*m*_/*k*_B_, inverse of mixing temperature. E1 and E2 were calculated for two MENM low frequency modes of RegX3 active and inactive states with Hessian matrix.

To detect hot spot residues, response of harmonic spring to perturbation in frequency of mode M was calculated as:

δω(M,n)=VMT.δH.VM.δH

Where VM represents the eigenvector of mode M at residue position n and δH is the observed change of Hessian matrix to the energy of elastic network. δω(M, n) indicates sensitivity of mode M to hot spot residues which illustrates evolutionary conservation and functional relevance [25].

## Supporting Information

S1 Fig Projection of clustered conformers on to the principal components obtained from 150 ns MD trajectories of RegX3. The contribution pattern and variance in the MD trajectories are presented in clusters with different colors in PC1, PC2. Cyan represents the first cluster followed by black, green, blue and red. Members from the respective cluster are shown with the same color. The clustering was made with 100 strides from MD trajectories that explain the behavior of conformational transition of RegX3.
(TIF)

S2 Fig Angular rotation of the effector domain (ED). (A). Original active state RegX3 (green) and simulated RegX3s (RD; grey, ED; orange) are aligned using core RD. A closer rotation of ED (174°) and 51.6° downward shift of helix α4 is shown. (B). Time series for rmsd difference in MD trajectories. (C). Superimposition of ED from MD conformers. Different colors correspond to the clusters made in S1 Fig. (D). RMSF representation of the ED in MD simulation.
(TIF)

S3 Fig Helix α1 outward shift. Structure alignment of rigid secondary structure elements of the RD of RegX3 (grey) and RegX3s (orange) is shown highlighting the 5.7° rotation and slight movement of N-terminal region of helix α1.
(TIF)

S4 Fig Projection of clustered conformers on to the principal components obtained from 5 ns MD trajectories of mutant RegX3. (A-H) Distinct contributions of mutants MD trajectories highlighting conformational paths are shown in different colors on PC planes.
(TIF)

S5 Fig Global scale effects on dimeric surface. (A-H) Impact of given mutation on dimeric interface are shown. Grey and orange colors designate 0 ns and the given mutant structure.
(TIF)
