# Supplementary figures and images for: Conformational Dynamics of Response Regulator RegX3 from Mycobacterium tuberculosis
Source: PLoS One. 2015 Jul 22;10(7):e0133389. doi: 10.1371/journal.pone.0133389 (PMC4511772; doi:10.1371/journal.pone.0133389)

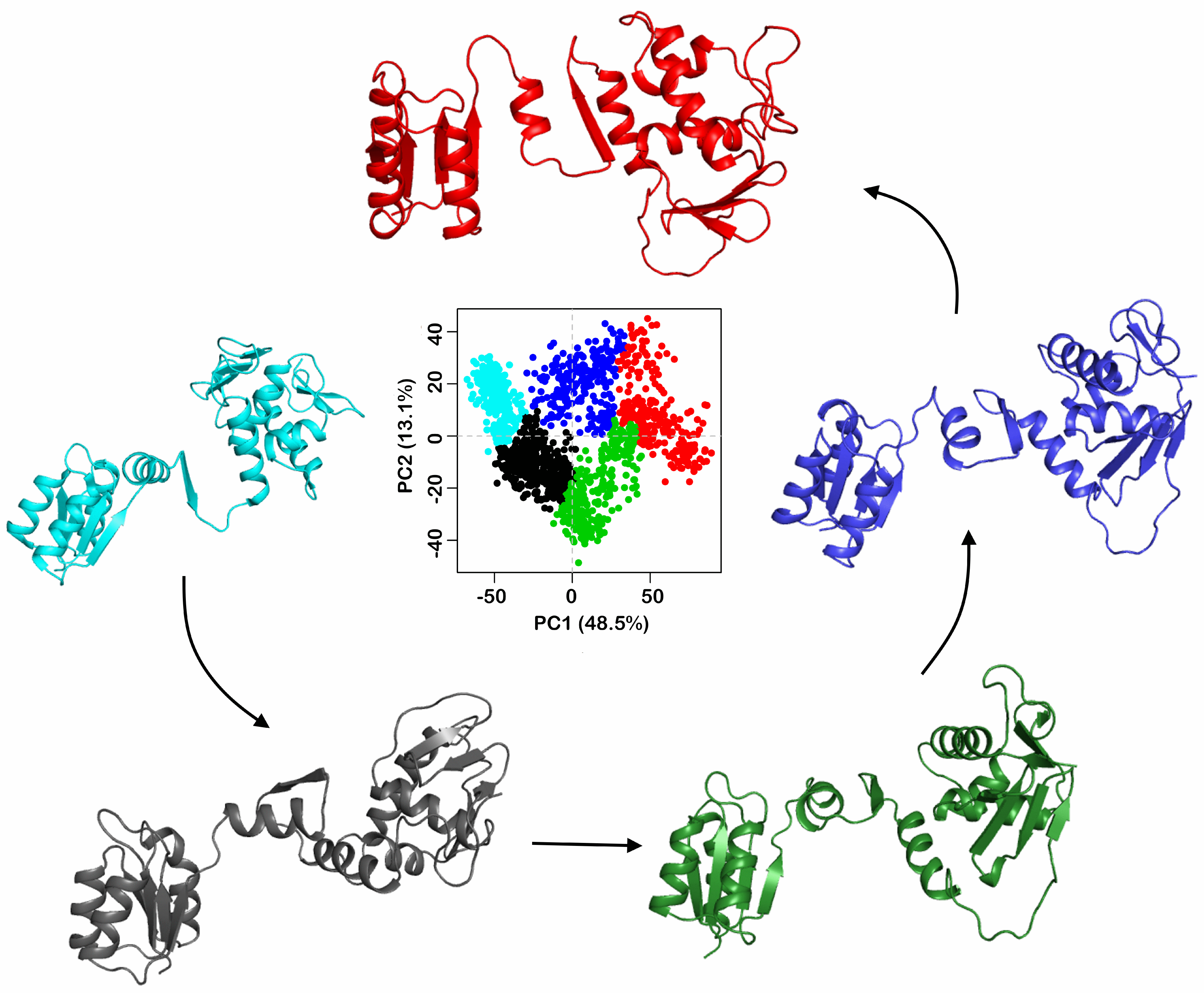

Supplement: S1 Fig — The contribution pattern and variance in the MD trajectories are presented in clusters with different colors in PC1, PC2. Cyan represents the first cluster followed by black, green, blue and red. Members from the respective cluster are shown with the same color. The clustering was made with 100 strides from MD trajectories that explain the behavior of conformational transition of RegX3. (TIF) [file pone.0133389.s001.tif]

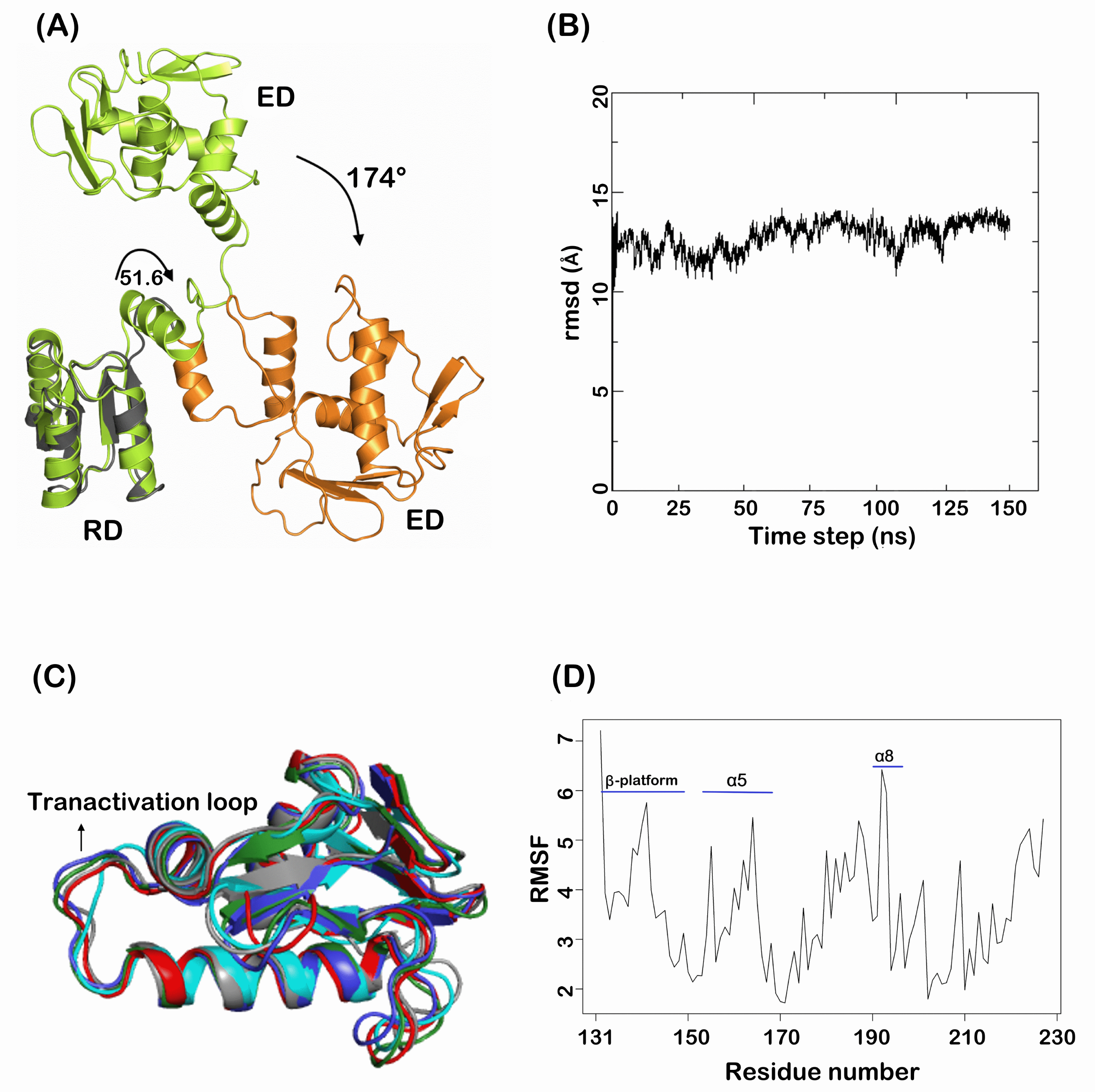

Supplement: S2 Fig — (A). Original active state RegX3 (green) and simulated RegX3s (RD; grey, ED; orange) are aligned using core RD. A closer rotation of ED (174°) and 51.6° downward shift of helix α4 is shown. (B). Time series for rmsd difference in MD trajectories. (C). Superimposition of ED from MD conformers. Different colors correspond to the clusters made in S1 Fig. (D). RMSF representation of the ED in MD simulation. (TIF) [file pone.0133389.s002.tif]

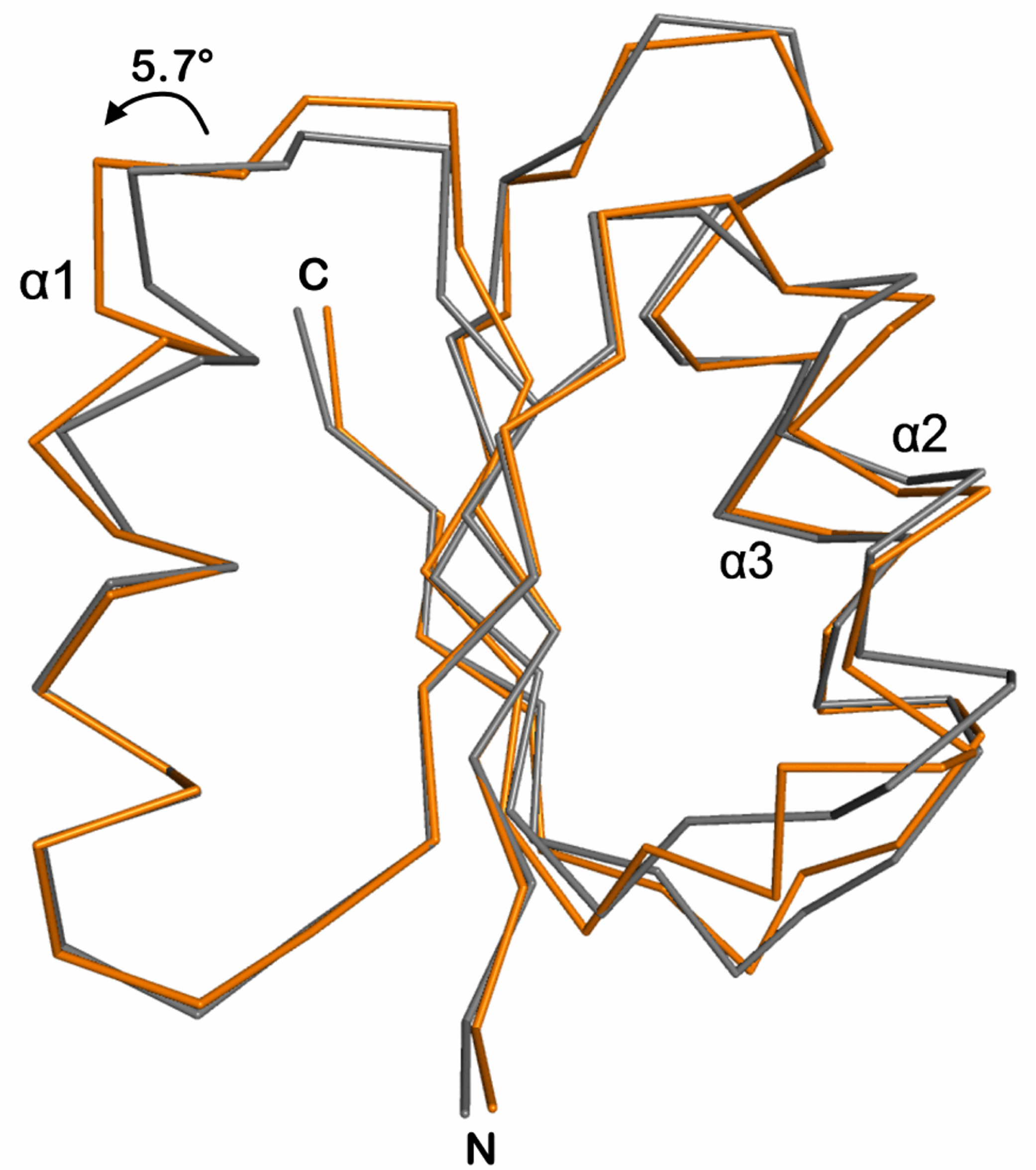

Supplement: S3 Fig — Structure alignment of rigid secondary structure elements of the RD of RegX3 (grey) and RegX3s (orange) is shown highlighting the 5.7° rotation and slight movement of N-terminal region of helix α1. (TIF) [file pone.0133389.s003.tif]

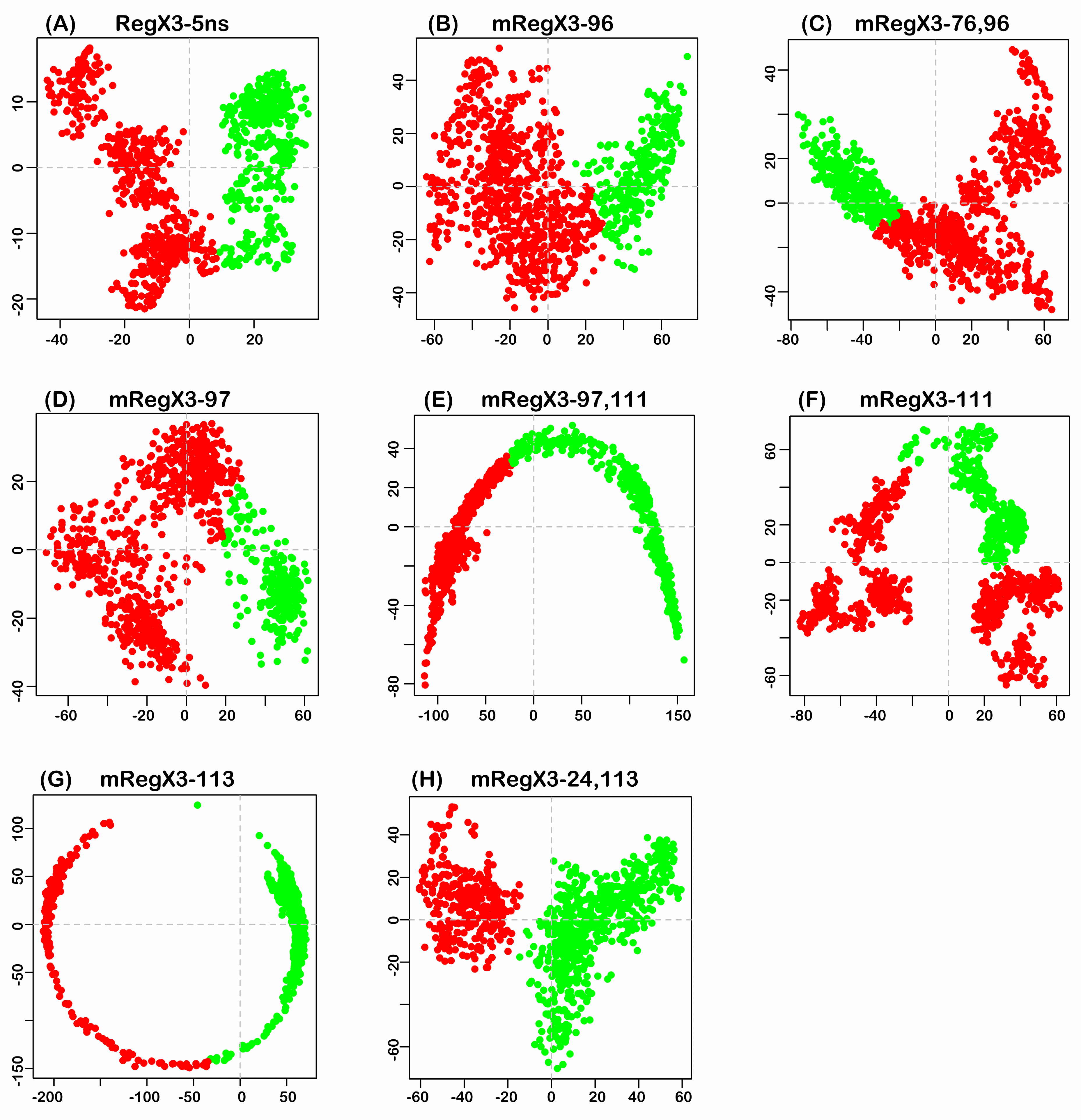

Supplement: S4 Fig — (A-H) Distinct contributions of mutants MD trajectories highlighting conformational paths are shown in different colors on PC planes. (TIF) [file pone.0133389.s004.tif]

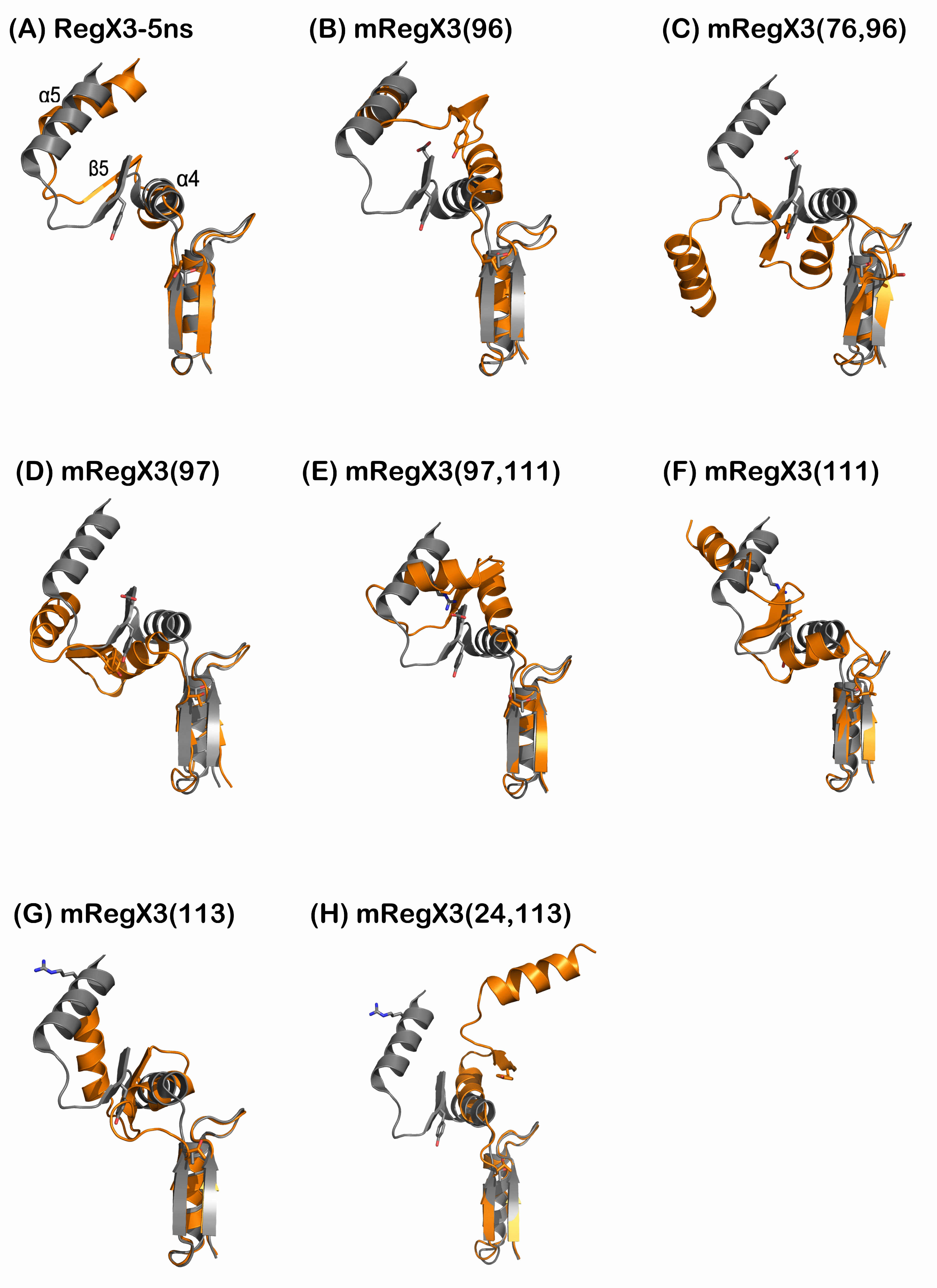

Supplement: S5 Fig — (A-H) Impact of given mutation on dimeric interface are shown. Grey and orange colors designate 0 ns and the given mutant structure. (TIF) [file pone.0133389.s005.tif]
